# Role of Vitamin D_3_ in Modulation of ΔNp63α Expression during UVB Induced Tumor Formation in SKH-1 Mice

**DOI:** 10.1371/journal.pone.0107052

**Published:** 2014-09-05

**Authors:** Natasha T. Hill, Gabriel H. Gracia-Maldonado, Mary K. Leonard, Amanda R. Harper, Kathleen L. Tober, Tatiana M. Oberyszyn, Madhavi P. Kadakia

**Affiliations:** 1 Department of Biochemistry and Molecular Biology; Boonshoft School of Medicine; Wright State University; Dayton, Ohio, United States of America; 2 Department of Pathology, The Ohio State University, Columbus, Ohio, United States of America; Rush University Medical Center, United States of America

## Abstract

ΔNp63α, a proto-oncogene, is up-regulated in non-melanoma skin cancers and directly regulates the expression of both Vitamin D receptor (VDR) and phosphatase and tensin homologue deleted on chromosome ten (PTEN). Since ΔNp63α has been shown to inhibit cell invasion via regulation of VDR, we wanted to determine whether dietary Vitamin D_3_ protected against UVB induced tumor formation in SKH-1 mice, a model for squamous cell carcinoma development. We examined whether there was a correlation between dietary Vitamin D_3_ and ΔNp63α, VDR or PTEN expression *in vivo* in SKH-1 mice chronically exposed to UVB radiation and fed chow containing increasing concentrations of dietary Vitamin D_3_. Although we observed differential effects of the Vitamin D_3_ diet on ΔNp63α and VDR expression in chronically irradiated normal mouse skin as well as UVB induced tumors, Vitamin D_3_ had little effect on PTEN expression *in vivo*. While low-grade papillomas in mice exposed to UV and fed normal chow displayed increased levels of ΔNp63α, expression of both ΔNp63α and VDR was reduced in invasive tumors. Interestingly, in mice fed high Vitamin D_3_ chow, elevated levels of ΔNp63α were observed in both local and invasive tumors but not in normal skin suggesting that oral supplementation with Vitamin D_3_ may increase the proliferative potential of skin tumors by increasing ΔNp63α levels.

## Introduction

1α,25-dihydroxyvitamin D_3_ (1,25(OH)_2_D_3_) has been investigated as an adjuvant to anti-cancer therapies. Upon binding to Vitamin D Receptor (VDR), 1,25(OH)_2_D_3_ induces expression of genes involved in apoptosis, differentiation and growth suppression while down regulating expression of genes that are involved in proliferation (reviewed in [1]). Keratinocytes synthesize 7-dehydrocholestrol, which is then converted to cholecalciferol by exposure to ultraviolet B (UVB) light between 280–320 nm. Intriguingly, these wavelengths of UVB are also the primary cause of skin cancer. Unlike keratinocytes, no other cell types can produce 1,25(OH)_2_D_3_ from 7-dehydrocholestrol and must rely on the sequential transport of cholecalciferol to the liver and kidneys to produce 25-hydroxyvitamin D_3_ and 1,25(OH)_2_D_3_, respectively. Due to the relative instability of 1,25(OH)_2_D_3_, dietary supplements commonly consist of cholecalciferol, also referred to as Vitamin D_3_ and rely on the conversion to 1,25(OH)_2_D_3_ by the liver and kidneys.

Severe Vitamin D_3_ deficiency, measured by serum 25-hydroxyvitamin D levels, or deletion of the VDR gene is associated with increased cancer risk [2], [3]. Although topical application of 1,25(OH)_2_D_3_ reduced UVB-induced tumor burden in the SKH-1 mouse model of squamous cell carcinoma [4], protective effects of dietary Vitamin D_3_ against the development of skin cancer has not been examined. This is an important study due to recent reports highlighting the frequency of Vitamin D_3_ deficiency, and its association with a myriad of disease states which has led to an increase in Vitamin D_3_ supplement intake by the general public [5].

On a cellular level, 1,25(OH)_2_D_3_, a downstream metabolite of Vitamin D_3_, exerts its biological function by binding the transcription factor VDR to control the expression of target genes. We have previously demonstrated that p63 inhibits cell invasion by directly regulating VDR and that both VDR and p63 are needed to inhibit cell invasion [6], [7]. The transcription factor p63 is essential for normal epidermal stratification and the proliferative potential of the epithelial stem cells [8], [9]. The *Tp63* gene can form several isoforms with contrasting functions, using alternate promoters and 3′ splicing. The TA isoforms (TAp63α, TAp63β and TAp63γ) have a full-length N-Terminal transactivation domain, whereas the ΔN isoforms (ΔNp63α, ΔNp63β and ΔNp63γ) have a unique truncated transactivation domain [10]. Our laboratory as well other researchers have previously shown that ΔNp63α is the only detectable p63 isoform expressed in the epidermis, specifically found in the proliferative basal layer [7], [11]–[15]. ΔNp63α is overexpressed in squamous cell carcinomas (SCC) and basal cell carcinomas (BCC) [11]–[13], [16], [17]. Contrary to its known roles in promoting epidermal differentiation, VDR levels, much like ΔNp63α, are also elevated in BCC and SCC [18], [19]. Through its ability to induce VDR, ΔNp63α could enhance 1,25(OH)_2_D_3_ signaling in non-melanoma skin cancers.

In cell culture systems, 1,25(OH)_2_D_3_ seems to have paradoxical pro-growth and pro-apoptotic functions. 1,25(OH)_2_D_3_ can prevent apoptosis of UV-irradiated keratinocytes in culture through the stabilization of ΔNp63α [20], or promote apoptosis through increased expression of the tumor suppressor phosphatase and tensin homolog deleted on chromosome 10 (PTEN) [21]. We have demonstrated that ΔNp63α negatively regulates PTEN expression and localization in keratinocytes to maintain normal growth rates. Moreover, the ratio of ΔNp63α to PTEN expression is significantly perturbed in human non-melanoma skin cancers [15].

In this study, we sought to delineate whether dietary Vitamin D_3_ offered any protection against UVB induced tumor formation and whether it preferentially induced expression of ΔNp63α, VDR, or PTEN *in vivo*. We fed SKH-1 hairless mice chow containing increasing concentrations of Vitamin D_3_ (cholecalciferol) and chronically exposed them to UVB light modeling the process of UV induced skin carcinogenesis in humans [14]. It has been shown that development of skin tumors in the SKH-1 hairless mice resemble UV induced squamous cell carcinomas in humans both morphologically as well at the molecular level [22].

Our results demonstrated that dietary Vitamin D_3_ offered no protection from UVB induced tumor formation and in fact increased tumor size at the highest dose tested. We observed differential effects of Vitamin D_3_ diet on ΔNp63α and VDR but not PTEN expression in chronically irradiated, but otherwise normal skin and in UVB induced tumors.

## Results

### Effects of dietary Vitamin D_3_ on epidermal structure

**Figure 1:**
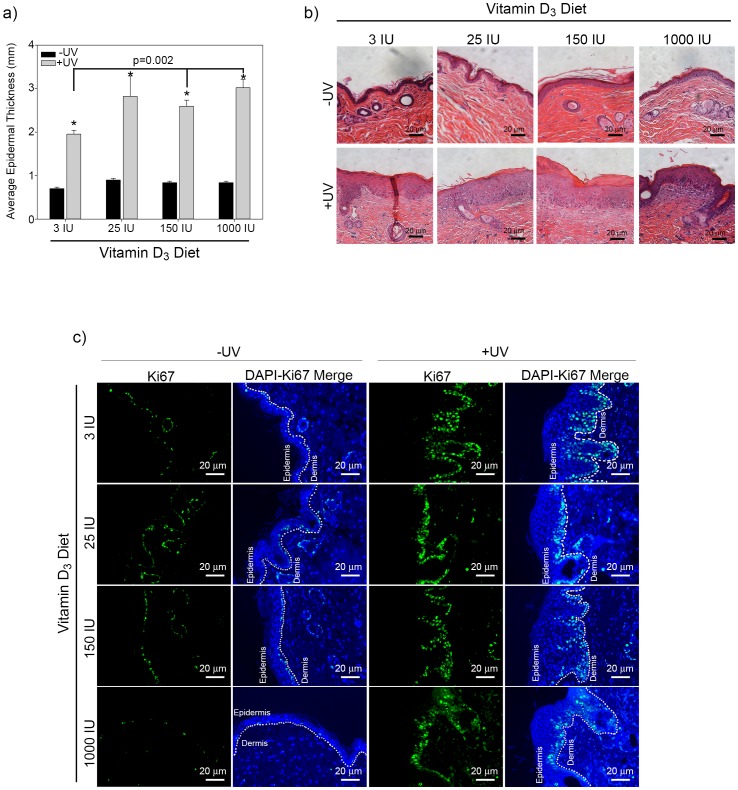
Dietary Vitamin D_3_ does not significantly alter epidermal thickening induced in response to UVB irradiation. (a) Male SKH-1 mice were fed diets with increasing concentrations of Vitamin D_3_ and irradiated thrice weekly for 25 weeks with UVB. The epidermal thickness from UV irradiated and unirradiated control mice are plotted. Error bars represent s.e.m. n = 15 UV exposed and n = 10 control unirradiated mice per treatment condition. (b) Representative images of irradiated and unirradiated skin after Haemotoxylin and Eosin (H&E) staining. Photos from SKH-1 mice fed standard or increasing concentration of vitamin D**_3_** chow were taken at a 10x magnification, scale bar  = 20 µm. (c) Ki67 staining in normal skin from irradiated and unirradiated skin obtained from mice fed diets with increasing concentration of Vitamin D_3_ as indicated. Ki67 images were taken at a 20x magnification, scale bar  = 20 µm.

To investigate the effect of increasing dietary Vitamin D_3_ on epidermal biology we first measured the skin thickness in SKH-1 hairless mice exposed to chronic UVB irradiation. Dietary Vitamin D_3_ alone did not alter the epidermal thickness of unirradiated mice at any dose tested indicating that dietary Vitamin D_3_ alone is insufficient to change epidermal proliferation. Chronic UVB exposure significantly increased epidermal thickness in all mice (Figure 1a &b). Interestingly, animals fed chow with higher concentrations of dietary Vitamin D_3_ displayed increased epidermal thickness in response to chronic UVB as compared to a standard (3 IU) diet (Figure 1a & b). To assess changes in proliferation, non-tumor dorsal skin sections were stained for Ki67. As shown is Figure 1c regardless of Vitamin D_3_ diet there was an increase in Ki67-positive cells in irradiated skin when compared to un-irradiated skin from SKH-1 mice.

**Figure 2:**
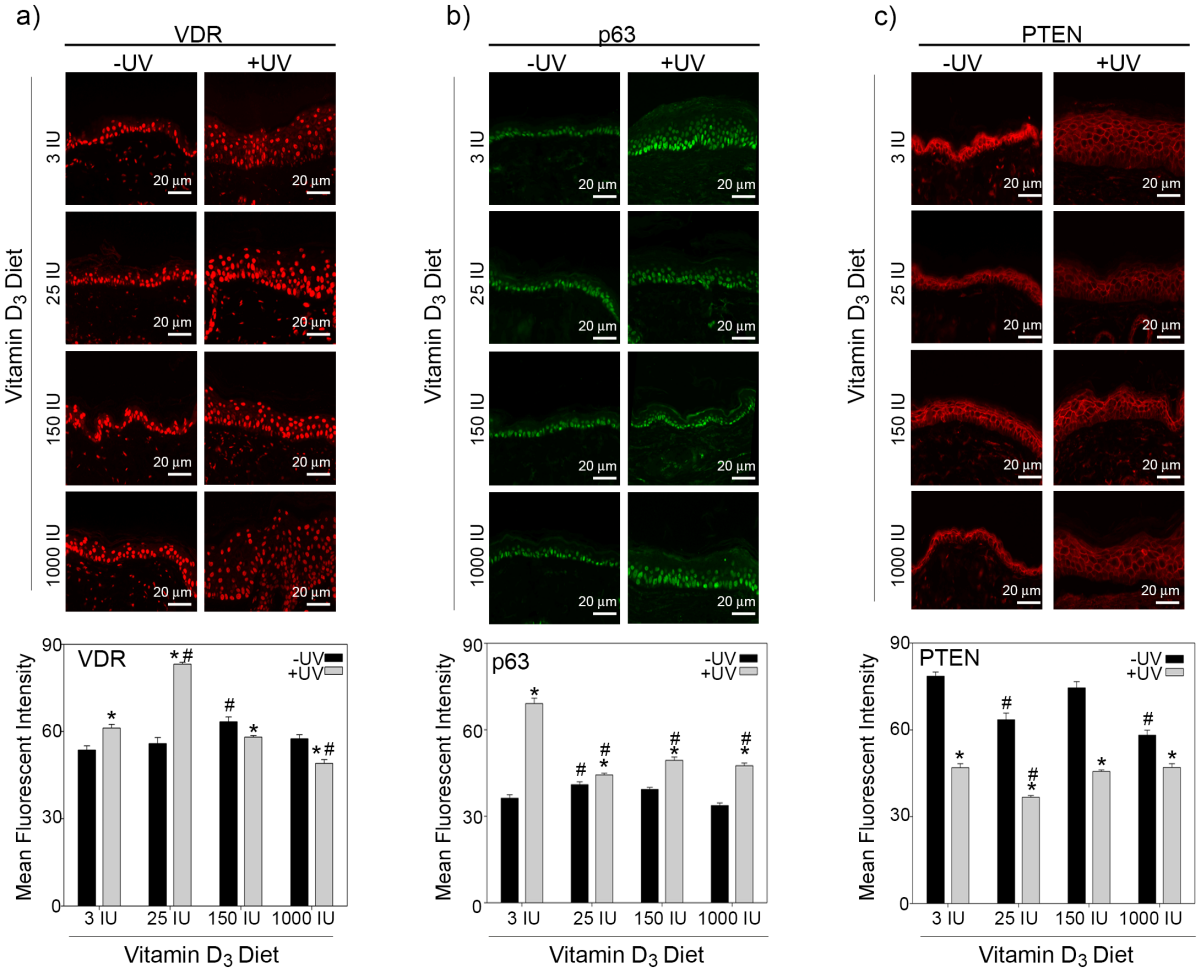
Dietary Vitamin D_3_ differentially affects VDR, ΔNp63α and PTEN levels in response to UVB. Top panels show representative images of (a) VDR, (b) ΔNp63α, or (c) PTEN staining in normal skin from irradiated and unirradiated skin obtained from mice fed diets with increasing concentration of Vitamin D_3_ as indicated were taken at a 20x magnification, scale bar  = 20 µm. Quantitation of (a) VDR, (b) ΔNp63α, or (c) PTEN staining from three animals per treatment condition is plotted in the lower panels. Y-axis represents the mean fluorescent intensity, normalized to background, in arbitrary units. Error bars represent standard error of mean. * = p≤0.05 compared to unirradiated skin; # = p≤0.05 compared to respective unirradiated or irradiated skin from mice fed 3 IU Vitamin D_3_.

Epidermal thickness is mediated by changes in keratinocyte proliferation and differentiation, both of which are regulated by VDR and ΔNp63α. 1,25(OH)_2_D_3_ has also been shown to stabilize both VDR and ΔNp63α [20], [23]. To determine whether the increase in epidermal thickness caused by increased dietary Vitamin D_3_ was the result of enhanced VDR or ΔNp63α expression, we stained skin tissues from UVB irradiated or control SKH-1 mice fed varying doses of dietary Vitamin D_3_ for VDR, ΔNp63α and their common transcriptional target PTEN. Since, ΔNp63α is the only detectable p63 isoform found in the epidermis we used a pan p63 antibody to detect ΔNp63α expression levels in the skin tissues [7], [11]–[15]. In unirradiated skin, increasing concentrations of dietary Vitamin D_3_ had little effect on the expression of VDR (Figure 2a, quantitated in lower panel). Lower doses of dietary Vitamin D_3_ significantly increased VDR expression in chronically UVB irradiated skin as compared to unirradiated skin (Figure 2a). Interestingly, the increase in VDR was not observed with higher concentrations of dietary Vitamin D_3_ in irradiated skin and in fact VDR was significantly down regulated in mice fed 1000 IU of Vitamin D_3_ diet compared to irradiated mice fed the standard (3 IU) diet (Figure 2a).

Similarly, Vitamin D_3_ diet did not drastically alter ΔNp63α expression in unirradiated skin (Figure 2b, quantitated in lower panel). In mice fed a standard diet of Vitamin D_3_, chronic exposure to UVB led to a significant increase in ΔNp63α expression in the epidermis as compared to unirradiated mice (Figure 2b). Contrary to previous reports in cultured keratinocytes treated with calcitriol and exposed to acute UV radiation [20], increasing concentrations of dietary Vitamin D_3_ led to a reduction in the ΔNp63α expression in response to chronic UVB exposure (Figure 2b).

Epidermal growth is also regulated by the tumor suppressor PTEN, which inhibits cell proliferation [24], [25]. Interestingly, increasing concentrations of dietary Vitamin D_3_ (25 and 1000 IU) significantly decreased PTEN expression in the epidermis of unirradiated mice as compared to mice fed a standard 3 IU Vitamin D_3_ diet (Figure 2c, quantitated in lower panel). Chronic exposure to UVB significantly reduced the expression of PTEN in the epidermis compared to unirradiated mice (Figure 2c). Increasing dietary Vitamin D_3_ in UVB irradiated mice did not further reduce PTEN levels.

### Dietary Vitamin D_3_ trends toward increased UVB-induced tumor development

We next wanted to determine whether dietary Vitamin D_3_ affects tumor formation, specifically tumor size and grade, in response to chronic UVB exposure. Representative images of the histology of the normal skin, papilloma, micro-invasive squamous cell carcinoma (MiSCC) and SCC are shown in Figure S1, as described previously [22]. Although increasing the amount of Vitamin D_3_ in the diet trended toward an increase in the average tumor area (Figure S2a) it was not statistically significant. Moreover, mice fed higher doses of dietary Vitamin D_3_ displayed a higher frequency of fully invasive squamous cell carcinomas (SCC) as compared to mice fed a standard diet (Figure S2b), but again this trend was not statistically significant. The increase in SCC in mice fed 1000 IU VD_3_ did not alter the frequency of papillomas, but rather correlated with a decrease in MiSCC as compared to the mice fed standard diet, suggesting that higher dietary Vitamin D_3_ may enhance tumor progression rather than tumor initiation (Figure S2b).

### Dietary Vitamin D_3_ differentially affects proteins involved in epidermal maintenance during tumor progression

**Figure 3:**
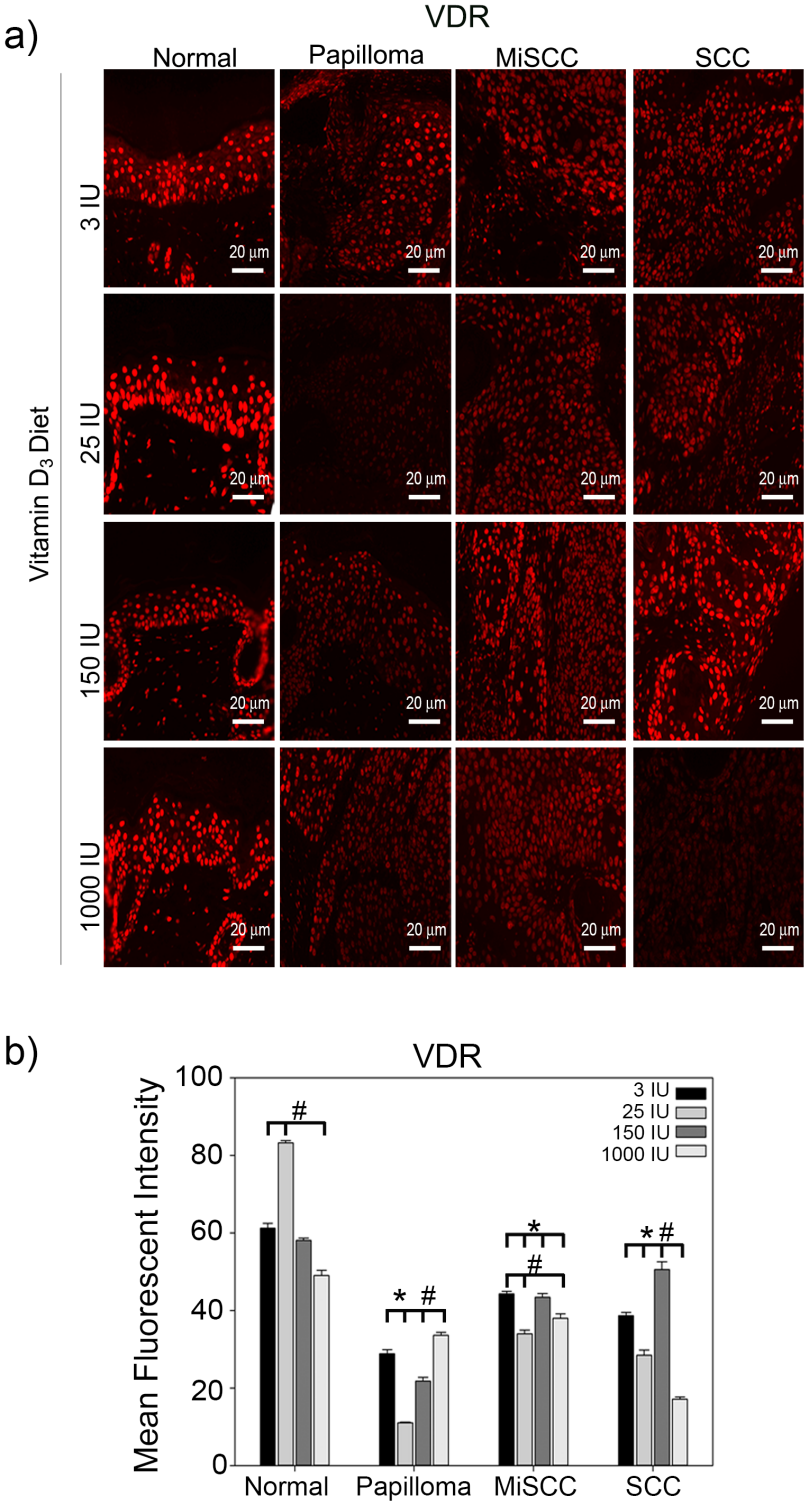
Effects of dietary Vitamin D_3_ on VDR expression during tumor progression. (a) Top panels show representative images taken at a 20x magnification, scale bar  = 20 µm of normal skin, benign papillomas, MiSCC and squamous cell carcinoma (SCC) from mice fed diets of increasing concentrations of Vitamin D_3_ stained for VDR. (b) Quantitation of VDR levels from three animals per treatment condition is plotted. Y-axis represents the mean fluorescent intensity, normalized to background, in arbitrary units. Error bars represent s.e.m. * = p≤0.05 compared to normal skin from the same diet; # = p≤0.05 compared to tissue of same tumor grade from mice fed 3 IU Vitamin D_3_.

VDR has been shown to inhibit cell invasion [7], a hallmark of tumor progression, and yet it has also been reported to be elevated in BCC and SCC [18], [19]. To determine whether there is a correlation between VDR expression, Vitamin D_3_ diet, and tumor grade, we determined VDR intensity in tumors of each grade from mice fed increasing doses of dietary Vitamin D_3_. VDR expression was significantly reduced in papillomas when compared to normal epidermal tissue regardless of dietary levels of Vitamin D_3_ (Figure 3). VDR levels were also significantly reduced in MiSCC and SCC as compared to normal epidermal tissue for all doses of dietary Vitamin D_3_ tested. Interestingly, VDR expression was significantly reduced in SCCs formed in mice fed a 1000 IU Vitamin D_3_ diet when compared to SCCs formed in mice fed a standard diet. The lack of VDR, which has tumor suppressive functions [3], in SCCs from mice fed 1000 IU Vitamin D_3_ diet (Figure 3b) may explain the trend toward increased frequency of SCC in animals on this diet (Figure S1b).

**Figure 4:**
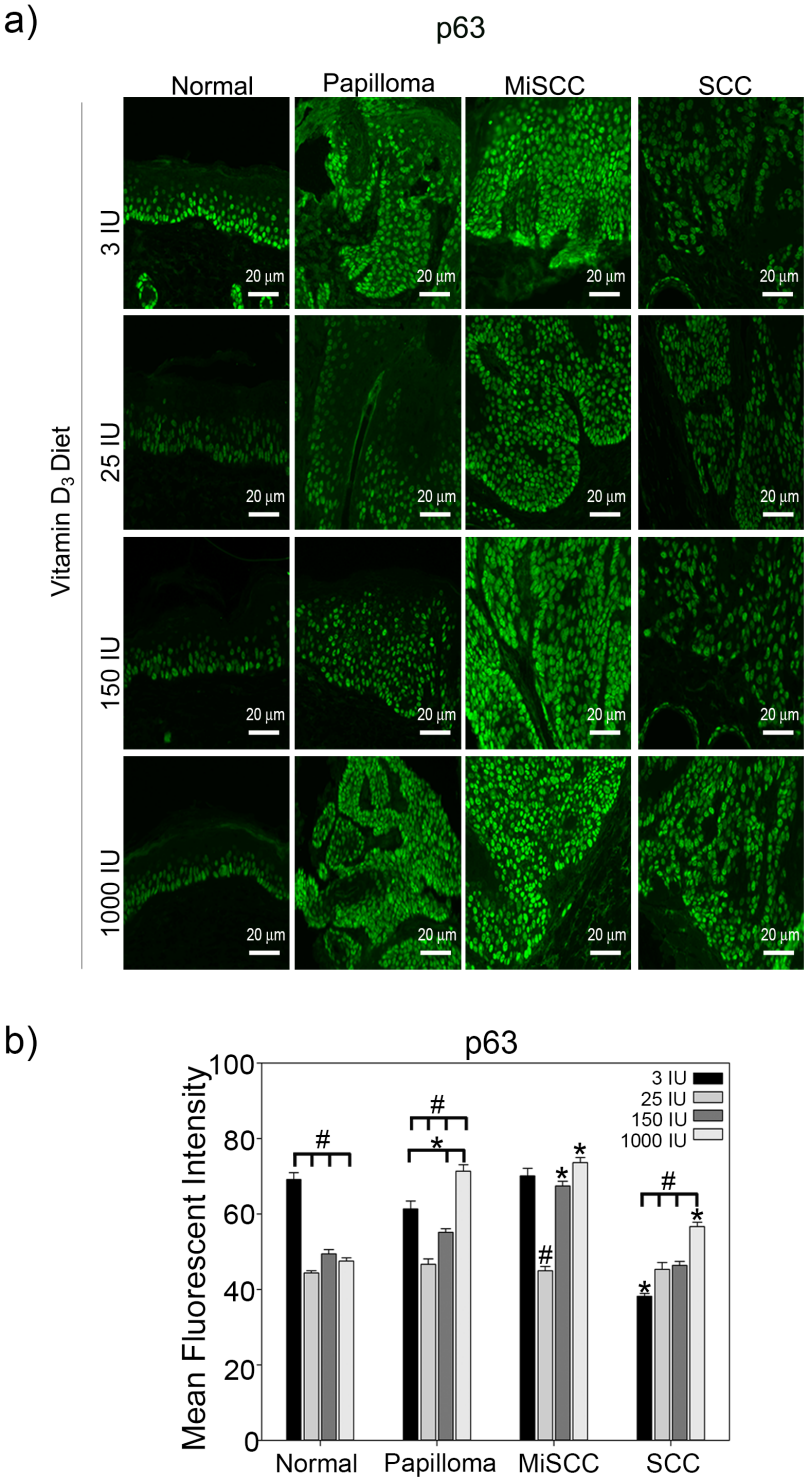
Effects of dietary vitamin D_3_ on ΔNp63α expression during tumor progression. (a) Top panels show representative images taken at a 20x magnification, scale bar  = 20 µm of normal skin, benign papillomas, MiSCC and SCC from mice fed diets of increasing concentrations of Vitamin D**_3_** stained for ΔNp63α. (b) Quantitation of ΔNp63α levels from three animals per treatment condition is plotted. Y-axis represents the mean fluorescent intensity, normalized to background, in arbitrary units. Error bars represent s.e.m. * = p≤0.05 compared to normal skin from the same diet; # = p≤0.05 compared to tissue of same tumor grade from mice fed 3 IU Vitamin D**_3_**.

**Figure 5:**
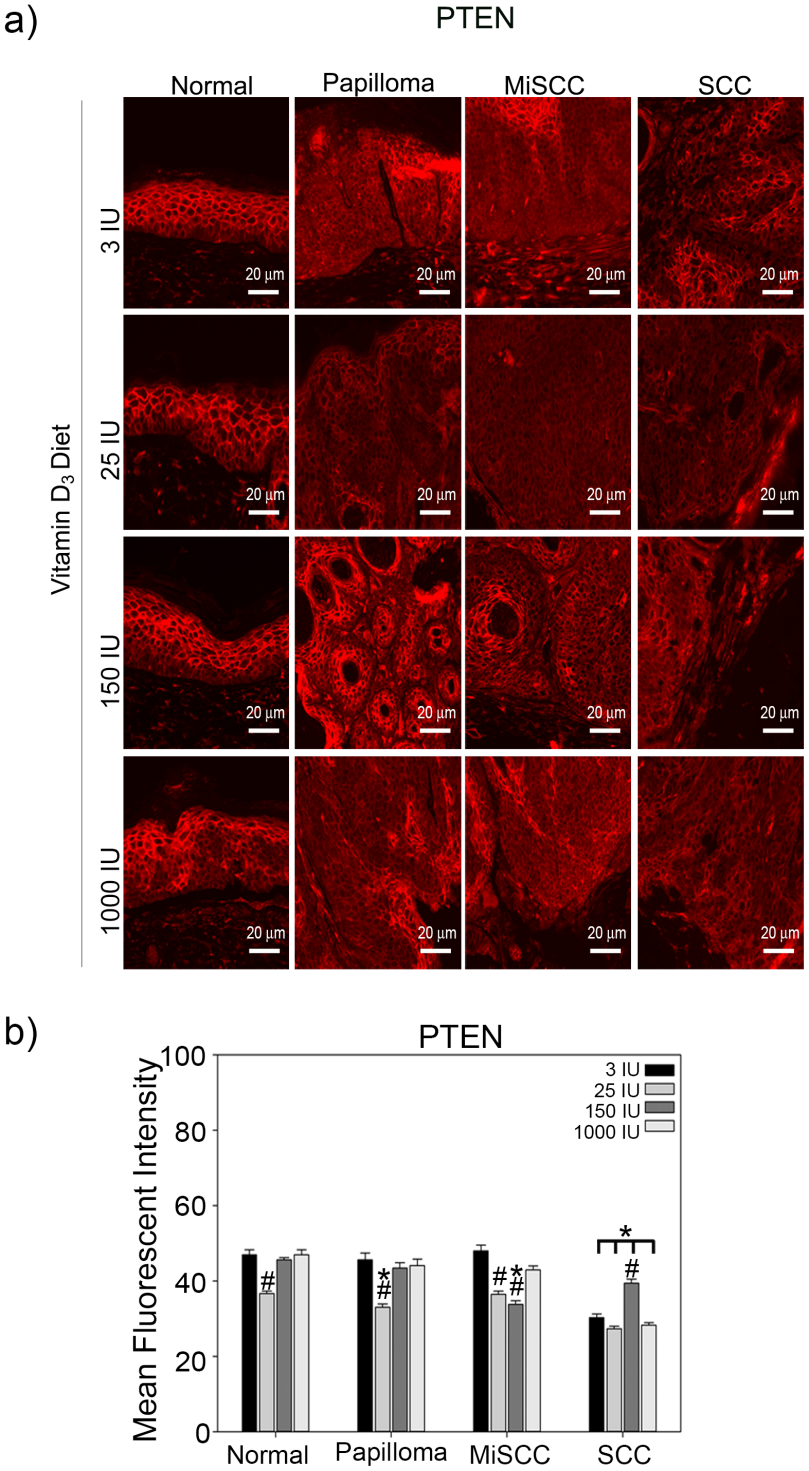
Effects of dietary Vitamin D_3_ on PTEN expression during tumor progression. (a) Top panels show representative images taken at a 20x magnification, scale bar  = 20 µm of normal skin, benign papillomas, MiSCC and SCC from mice fed diets of increasing concentrations of Vitamin D**_3_** stained for PTEN. (b) Quantitation of PTEN levels from three animals per treatment condition is plotted. Y-axis represents the mean fluorescent intensity, normalized to background, in arbitrary units. Error bars represent s.e.m. * = p≤0.05 compared to normal skin from the same diet; # = p≤0.05 compared to tissue of same tumor grade from mice fed 3 IU Vitamin D_3_.

ΔNp63α, known to increase the proliferation of epidermal keratinocytes, was significantly down regulated in normal epidermal tissue at all doses of dietary Vitamin D_3_ when compared to mice fed a standard diet (Figure 4). Similar to VDR, ΔNp63α expression was also increased in a dose dependent manner in papillomas fed increasing doses of vitamin D_3_ chow. However, unlike VDR, ΔNp63α expression levels were also increased in both MiSCCs and SCCs (Figure 4b) with increasing doses of Vitamin D_3_ diet. Interestingly, papillomas and MiSCC from mice on the higher dietary Vitamin D_3_ (150 IU and 1000 IU) expressed significantly more ΔNp63α than normal epidermal tissue from mice of the same diet (Figure 5b). Loss of p63 has been associated with increased cell invasion in urothelial and bladder cancers [26], [27]. Our results also demonstrated a significant reduction in ΔNp63α expression in SCCs compared to MiSCC and normal epidermal tissues from mice fed a standard diet (Figure 4b). However, SCCs from mice fed increasing concentrations of Vitamin D_3_ diet exhibited a dose dependent increase in ΔNp63α expression levels suggesting that dietary Vitamin D_3_ enhances the proliferative nature of SCC by preventing the down regulation of ΔNp63α (Figure 4b).

To investigate if dietary Vitamin D_3_ leads to a reduction in the expression of tumor suppressor PTEN, we measured the expression of PTEN by immunofluorescence in normal skin and tumors from UVB irradiated mice fed each of the Vitamin D_3_ diets. Increasing the concentration of Vitamin D_3_ in the diet did not have consistent trends on the expression of PTEN between tumor types (Figure 5). Consistent with previous reports [28], PTEN was significantly reduced in UVB induced SCC compared to normal skin independent of the Vitamin D_3_ diet (Figure 5), suggesting that dietary Vitamin D_3_ does not increase the tumor size or burden by augmenting UVB mediated degradation of PTEN.

**Figure 6:**
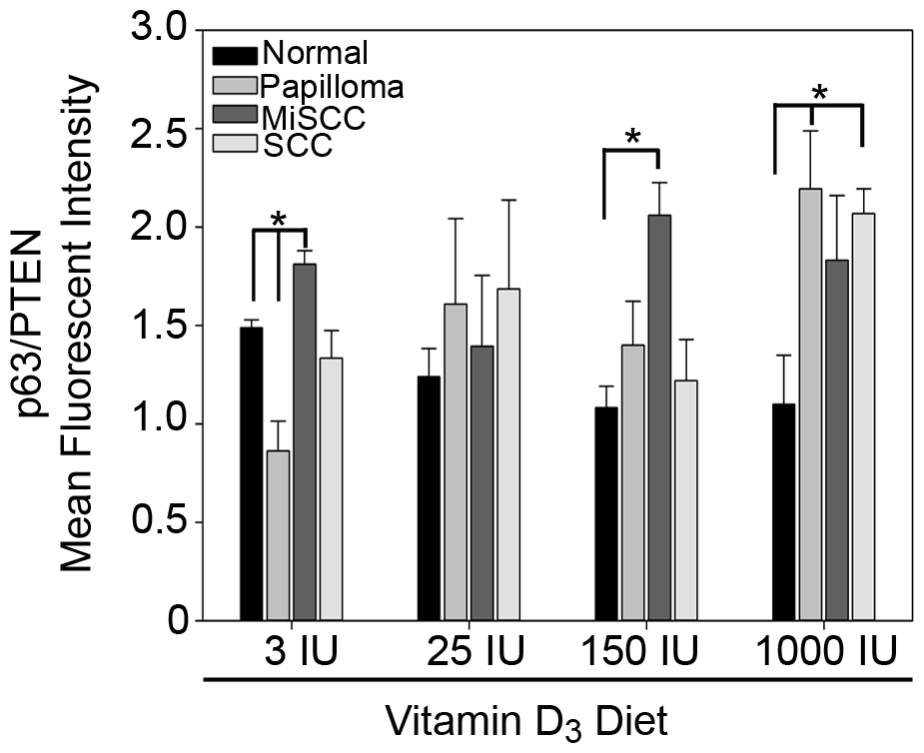
Dietary Vitamin D_3_ alters the ratio of ΔNp63α to PTEN during tumor progression. The average ratio of ΔNp63α fluorescence intensity to PTEN fluorescence intensity from normal skin, benign papillomas, MiSCC, and SCC from mice fed diets of increasing concentrations of Vitamin D_3_ as indicated is plotted. Error bars represent standard error of mean from three animals per treatment condition. * = p≤0.05 compared to unirradiated skin.

We have previously demonstrated that the ratio of ΔNp63α to PTEN is critical for mediating keratinocyte proliferation and that this ratio is significantly perturbed in human BCC and SCC [15]. To determine if perturbation of the balance between ΔNp63α and PTEN by dietary Vitamin D_3_ was contributing to the increase in tumor size and SCC frequency, we calculated the ratio of ΔNp63α to PTEN fluorescence intensity in normal skin and tumors from UVB irradiated mice fed each of the Vitamin D_3_ diets. Mice fed a diet of 1000 IU Vitamin D_3_ displayed consistently higher ratios of ΔNp63α to PTEN, indicative of an increased proliferation potential, in all tumor types as compared to normal skin (Figure 6). Taken together, these studies suggest that increased dietary Vitamin D_3_ may enhance UVB induced tumor formation and progression, at least at supra-physiologic doses, by decreasing the expression of VDR while increasing the ΔNp63α to PTEN ratio.

## Discussion

1,25(OH)_2_D_3_ has been investigated as an adjuvant to anti-cancer therapies because of its growth suppressive and pro-differentiation properties. Although the association of Vitamin D_3_ consumption and serum 25-hydroxyvitamin D with the prevention of a wide range of cancers has been widely studied [29], evidence supporting the role of 1,25(OH)_2_D_3_ in protecting against skin cancer is often conflicting [30]–[32]. In this study we demonstrate that increased consumption of dietary Vitamin D_3_ in the SKH-1 mouse model of squamous cell carcinoma does not protect against UVB-induced tumor formation (Figure S1). Moreover, supra-physiologic levels (1000 IU) of dietary Vitamin D_3_ may actually promote epidermal proliferation and tumor formation as evidenced by increased epidermal thickness and Ki67 staining (Figure 1) and dose-dependent trends toward larger, more aggressive tumor development (Figure S2).

The enhanced proliferation and tumor development in UVB irradiated mice fed 1000 IU Vitamin D_3_ may be related to the stabilization of the ΔNp63α (Figure 4), which is often overexpressed in human non-melanoma skin cancers [11]–[13], [16], [17]. Numerous models of acute UVB irradiation have demonstrated that ΔNp63α must be down regulated to allow for apoptosis in the epidermis [33]–[35]. It has been previously shown that ablation of the basal layer cells of the interfollicular epidermis comprising of mutant p53 and p63-positive cells led to a significant delay in the onset of tumor formation in SKH-1 mice, suggesting that ΔNp63α likely contributed to tumor formation [36]. Our studies show that, unlike acute UVB exposure, ΔNp63α levels were significantly higher in chronically UVB irradiated skin (Figure 2b) potentially predisposing skin to tumor development. While we did not observe an increase in ΔNp63α levels in response to increased dietary Vitamin D_3_ in normal skin, we found that dietary Vitamin D_3_ was able to limit the down regulation of ΔNp63α during tumor progression (Figure 4). The sustained expression of ΔNp63α by dietary Vitamin D_3_ could contribute to the proliferation and expansion of UVB induced tumors.

Interestingly, the increase in ΔNp63α expression did not correlate with increased expression of VDR, a direct transcriptional target of p63 (Figures 3–4) [6]. This suggests that dietary Vitamin D_3_, at least in the context of concomitant UVB irradiation, may enhance the oncogenic properties of ΔNp63α by increasing the ratio of ΔNp63α to PTEN (Figure 6), rather than altering its tumor suppressive attributes, namely induction of VDR.

Unlike previous studies conducted in 1,25(OH)_2_D_3_ deficient rats, we did not observe an increase in epidermal VDR expression in response to increased dietary Vitamin D_3_ (Figures 2a and 3) [37]. This could be attributed to the inherent differences between rats and SKH-1 mice and/or the differences in experimental approach. In the studies conducted by Zineb et al., VDR expression was measured in Wistar rats that were kept in the dark, preventing the cutaneous production of 1,25(OH)_2_D_3_, and fed a diet lacking Vitamin D_3_ to induce 1,25(OH)_2_D_3_ deficiency before re-supplementation of dietary Vitamin D_3_ [37]. To better mimic the environmental conditions experienced by humans, our studies utilized a hairless mouse strain chronically exposed to UVB without inducing 1,25(OH)_2_D_3_ deficiency prior to dietary Vitamin D_3_ supplementation. It is important to note that while UVB is the most common cause of non-melanoma skin cancers and its use as a carcinogen is most physiologically relevant, the ability of keratinocytes in the epidermis to generate 1,25(OH)_2_D_3_ in response to UVB can confound the interpretation of how dietary Vitamin D_3_ affects tumor formation.

Our results suggest that increased dietary Vitamin D_3_ may enhance UVB induced tumor formation and progression (Figure S2) by decreasing the expression of VDR in the epidermis (Figure 3) while increasing ΔNp63α (Figure 4). The deleterious effects of dietary Vitamin D_3_ observed in this study are consistent with previous epidemiological studies showing that the risk for non-melanoma skin cancers was positively correlated with increasing serum 25-hydroxyvitamin D levels [30]. The U.S. Preventive Services Task Force has reported that there is insufficient data to support Vitamin D_3_ supplementation as a cancer prevention method [38]. However, more efficient delivery of 1,25(OH)_2_D_3_ to keratinocytes may also be critical to generating protective rather than deleterious effects with regard to UVB induced skin cancer.

A study by Dixon *et al*. demonstrated that topical application of 1,25(OH)_2_D_3_ led to a reduction in the development and size of UV-induced tumors in the SKH-1 mouse model of squamous cell carcinoma [4]. In contrast to our data obtained with dietary Vitamin D_3_ (Figure S2), topical 1,25(OH)_2_D_3_ led to a reduction in the incidence and progression of UV induced tumors [4]. Aside from choice and route of delivery of vitamin D, there were differences in the light source, UV exposure protocol, and sex of mice used in our study compared to the topical calcitriol study. Exposure of keratinocytes to UVB compared to solar simulated light can alter signaling pathways in the skin [39], [40]. Additionally, our lab has demonstrated significant differences in the response to UV light between the sexes [41] and also in response to treatment [42]. Topical application of the active Vitamin D_3_ metabolite 1,25(OH)_2_D_3_ allows for direct activation of VDR and its downstream effects in the skin. In contrast, the dietary Vitamin D_3_ used in our study, must be absorbed by the intestines, converted by liver and the kidney to 1,25(OH)_2_D_3_ and shuttled back through the blood stream to the tumor site where it has to reach critical levels to inhibit tumor progression.

Xenograft mice models of breast cancer have shown that dietary vitamin D_3_ inhibited tumor formation in breast fat pad, metastases to the lungs and reduced tumor size [43]. In this study they observed that mice fed diets of up to 5000 IU/kg dietary vitamin D_3_ had elevated 25(OH)D_3_ serum levels but no hypercalcemia as evidenced by lack of increased calcium levels in serum. [43]. Moreover, mice fed 5000 IU/kg of dietary vitamin D_3_ showed a reduction in the number and size of breast tumors. Differences in the effects of dietary Vitamin D_3_ supplementation in the two studies may be attributed to a 5 fold higher dose used in the breast cancer xenograft model when compared to the 1000 IU/kg used in our study as well as the tumor type being studied.

The current studies did not specifically examine the role of interfollicular vs follicular cells and Vitamin D_3_ supplementation in SCC formation. However, it has previously been shown that while removal of the interfollicular epidermis by abrasion in CD-1 haired mice decreased the quantity of papilloma developed by half, it did not delay or stop the development of papillomas [44]. Similarly, CO_2_ laser ablation of the interfollicular epidermis of hairless mice did not delay or stop the development of tumors, suggesting that a pool of cells deep in the hair follicle might be responsible for the SCC development [45]. UV-induced ablation of the epidermal basal layer in hairless mice further showed SCC originated from the interfollicular epidermis which was being repopulated from the hair follicle [36]. These studies suggest that the decrease in hair follicles in our hairless mice, observed as they age, did not impact tumor development in our study.

These studies demonstrate the complexity of Vitamin D_3_ supplementation and suggest the necessity for additional studies to determine whether dietary Vitamin D_3_ or topical 1,25(OH)_2_D_3_ are viable therapeutic options since the application of 1,25(OH)_2_D_3_ to un-irradiated normal hairless mouse skin results in dose and time dependent increases in mitosis and hyperplasia [46]. Taken together these studies demonstrate that Vitamin D_3_ may have differing effects depending on the target organ and mode of delivery. In the case of non-melanoma skin cancers it may be detrimental at high levels because of its ability to stabilize ΔNp63α levels and increase, rather than prevent, UVB induced tumors.

## Materials and Methods

### Animal Treatments

Male SKH-1 hairless mice were obtained from Charles River Laboratories (Wilmington MA). Male SKH-1 mice were housed in the vivarium at The Ohio State University according to the requirements established by the American Association for Accreditation of Laboratory Animal Care. The Ohio State University Institutional Animal Care and Use Committee approved all procedures before the initiation of any studies (Protocol Number: 2010A00000083) and all efforts were made to minimize suffering. Four week old animals were assigned to different diets consisting of either standard chow with only 3 IU/kg Vitamin D_3_ (8640 Teklad 22/5 Diet, Harlan Laboratories, Madison, WI), or AIN93G diet modified to contain 25 IU/kg, 150 IU/kg, or 1000 IU/kg Vitamin D_3_ (Research Diets, New Brunswick, NJ). The concentrations of Vitamin D_3_, in the form of cholecalciferol, was based on the study by Fleet *et al*. demonstrating that the dietary Vitamin D_3_ concentrations needed for modeling human borderline deficiency (25–40 nmol/L) average (50–60 nmol/L) and optimal (80–100 nmol/L) serum 25-hydroxyvitamin D concentrations as defined by NRC are 25–50, 100, and 400 IU Vitamin D_3_/kg diet in growing rodents [47]. Twenty-five mice were assigned to each diet. Fifteen mice per diet were dorsally exposed to 2240 J/m^2^ UVB, previously determined to be to one minimal erythemic dose, 3 times weekly for a total of 25 weeks. UVB dose was calculated using a UVX radiometer and UVB sensor (UVP, Upland, CA) and delivered using Philips TL 40W/12 RS SLV UVB broadband bulbs emitting 290–315 nm UVB light (American Ultraviolet Company, Lebanon, as previously described [48]. Ten mice per diet served as age matched, unirradiated controls. All mice were sacrificed by CO_2_ inhalation.

### Quantitation of epidermal thickness

Epidermal morphology was analyzed using the Accustain trichrome stain (Masson) kit according to manufacturer's instructions (Sigma-Aldrich, St. Louis, MO). Epidermal thickness was measured using ImageJ software at a magnification of 10x in all tissue samples. Dorsal skin morphology was examined using H&E staining and visualized/imaged using a Leica CTR 6000 Microscope (Leica Microsystems, Wetzlar, Germany) and ImagePro 6.2 software (Media Cybernetics, Bethesda, MD).

### Tumor development and grade

Neoplastic lesions located on the dorsal skin measuring greater than 1 mm in size were counted and measured (length × width). Tumors were measured using digital calipers throughout the duration of the study. Tumor grade was determined from hematoxyliln and eosin (H&E)-stained sections of tumors isolated from UVB irradiated mice graded in a blinded manner by a board certified veterinary pathologist as previously described [48]. Briefly, papillomas were exophytic tumors (tumors that grow outward from the originating epithelium) that showed no invasion of the stroma [22]. MiSCCs were distinguished by the depth of penetration into the dermis [22]. Only tumors that invaded the panniculus carnosus were classified as fully invasive SCCs [22]. Average tumor percentages were calculated using the total number of graded tumors per treatment group.

### Antibodies

PTEN, VDR, Ki67 and p63 antibodies were used to conduct immunofluorescence staining. Pan p63 (clone: 4A4) used to detect ΔNp63α, VDR (clone: 9A7) and PTEN (#9552) antibodies were purchased from (Santa Cruz, CA, USA), (Thermo-Scientific, Fremont, CA) and Cell Signaling (Danvers, MA, USA) respectively. Ki67 (clone: SP6) antibody was purchased from abcam (Cambridge, MA, USA).

### Immunofluorescence

Tumors excised from dorsal skin as well as non-tumor dorsal skin were formalin fixed, paraffin-embedded and stained for p63, VDR and PTEN as previously described [7], [15]. Ki67 staining was preformed analogous to previously described staining of p63 [7], [15]. For detection of VDR, paraffin was removed by four 10 minute washes in Histo-Clear (National Diagnostics, Atlanta, GA) and rehydrated in graded series of alcohols with a final wash in distilled water. After rehydration slides were incubated at 37°C for 20 minutes at 60°C in 2 N HCl. Slides were neutralized with 3 washes of 0.1 M sodium borate buffer (pH 8.5), followed by three washes in PBS. Tissues were blocked for 3 hours with 5% normal goat serum followed by overnight incubation with anti-VDR at 4°C (clone 9–A7, Thermo-Scientific, Fremont, CA). Excess primary antibody was removed with three consecutive washes in PBS followed by incubation with AlexaFluor 568 goat anti-rat antibody for 1 hour at room temperature. Excess secondary was removed with three consecutive 5 min washes in PBS prior to mounting with Vecta-Shield plus DAPI Mounting Media (Vector Laboratories, Burlingame, CA). Cells were visualized and imaged using a Leica CTR 6000 Microscope (Leica Microsystems, Wetzlar, Germany) and ImagePro 6.2 software (Media Cybernetics, Bethesda, MD). Mean fluorescence intensity for each tissue sample was calculated using ImagePro 6.2 software after normalization for background intensity. Multiple measurements (at least 5), all of the same size, were taken of the epidermal tissue for each tissue sample. Average mean fluorescence intensity was calculated as previously described [15].

### Statistics

Differences in mean fluorescence intensities were analyzed by one-way ANOVA followed by pairwise multiple comparison testing (Tukey test method, SigmaPlot 12, Dundas Software).

## Supporting Information

Figure S1 **SKH-1 mice skin following UVB induced tumor development.** SKH-1 mice fed chow with increasing concentration of Vitamin D_3_ were irradiated thrice weekly for 25 weeks with UVB. Tumor excised from dorsal skin as well as non-tumor (normal) dorsal skin were formalin fixed, paraffin embedded and subjected to H&E staining. Representative images of a normal skin, papilloma, MiSCC and SCC were taken at a 20x magnification. Scale bar  = 20 µm.
(PSD)

Figure S2 **Effect of dietary Vitamin D_3_ on tumor development.** (a) The average tumor area per mouse is plotted after 25 weeks of thrice weekly irradiation in mice fed diets with increasing amounts of Vitamin D**_3_**. Error bars represent s.e.m. (b) The distribution of premalignant papillomas and malignant microinvasive squamous cell carcinomas (MiSCC) and malignant SCC is plotted. Error bars represent s.e.m.; n = 15 mice per treatment condition.
(PSD)

## References

[1] Deeb KK, Trump DL, Johnson CS (2007) Vitamin D signalling pathways in cancer: potential for anticancer therapeutics. Nat Rev Cancer 7: 684–700. 17721433 10.1038/nrc2196

[2] Gandini S, Boniol M, Haukka J, Byrnes G, Cox B, et al (2011) Meta-analysis of observational studies of serum 25-hydroxyvitamin D levels and colorectal, breast and prostate cancer and colorectal adenoma. Int J Cancer 128: 1414–1424. 20473927 10.1002/ijc.25439

[3] Zinser GM, Sundberg JP, Welsh J (2002) Vitamin D(3) receptor ablation sensitizes skin to chemically induced tumorigenesis. Carcinogenesis 23: 2103–2109. 12507934 10.1093/carcin/23.12.2103

[4] Dixon KM, Norman AW, Sequeira VB, Mohan R, Rybchyn MS, et al (2011) 1alpha,25(OH)(2)-vitamin D and a nongenomic vitamin D analogue inhibit ultraviolet radiation-induced skin carcinogenesis. Cancer Prev Res (Phila) 4: 1485–1494. 21733837 10.1158/1940-6207.CAPR-11-0165

[5] Scragg R (2011) Vitamin D and public health: an overview of recent research on common diseases and mortality in adulthood. Public Health Nutr 14: 1515–1532. 21729467 10.1017/S1368980011001455

[6] Kommagani R, Caserta TM, Kadakia MP (2006) Identification of vitamin D receptor as a target of p63. Oncogene 25: 3745–3751. 16462763 10.1038/sj.onc.1209412

[7] Kommagani R, Leonard MK, Lewis S, Romano RA, Sinha S, et al (2009) Regulation of VDR by deltaNp63alpha is associated with inhibition of cell invasion. J Cell Sci 122: 2828–2835. 19622632 10.1242/jcs.049619 PMC2724606

[8] Mills AA, Zheng B, Wang XJ, Vogel H, Roop DR, et al (1999) p63 is a p53 homologue required for limb and epidermal morphogenesis. Nature 398: 708–713. 10227293 10.1038/19531

[9] Yang A, Schweitzer R, Sun D, Kaghad M, Walker N, et al (1999) p63 is essential for regenerative proliferation in limb, craniofacial and epithelial development. Nature 398: 714–718. 10227294 10.1038/19539

[10] Yang A, Kaghad M, Wang Y, Gillett E, Fleming MD, et al (1998) p63, a p53 homolog at 3q27–29, encodes multiple products with transactivating, death-inducing, and dominant-negative activities. Mol Cell 2: 305–316. 9774969 10.1016/s1097-2765(00)80275-0

[11] Bircan S, Candir O, Kapucoglu N, Baspinar S (2006) The expression of p63 in basal cell carcinomas and association with histological differentiation. J Cutan Pathol 33: 293–298. 16630179 10.1111/j.0303-6987.2006.00436.x

[12] Choi HR, Batsakis JG, Zhan F, Sturgis E, Luna MA, et al (2002) Differential expression of p53 gene family members p63 and p73 in head and neck squamous tumorigenesis. Hum Pathol 33: 158–164. 11957139 10.1053/hupa.2002.30722

[13] Di Como CJ, Urist MJ, Babayan I, Drobnjak M, Hedvat CV, et al (2002) p63 expression profiles in human normal and tumor tissues. Clin Cancer Res 8: 494–501. 11839669

[14] Koster MI, Kim S, Mills AA, DeMayo FJ, Roop DR (2004) p63 is the molecular switch for initiation of an epithelial stratification program. Genes Dev 18: 126–131. 14729569 10.1101/gad.1165104 PMC324418

[15] Leonard MK, Kommagani R, Payal V, Mayo LD, Shamma HN, et al (2011) DeltaNp63alpha regulates keratinocyte proliferation by controlling PTEN expression and localization. Cell Death Differ 18: 1924–1933. 21637289 10.1038/cdd.2011.73 PMC3214913

[16] Hibi K, Trink B, Patturajan M, Westra WH, Caballero OL, et al (2000) AIS is an oncogene amplified in squamous cell carcinoma. Proc Natl Acad Sci U S A 97: 5462–5467. 10805802 10.1073/pnas.97.10.5462 PMC25851

[17] Sakiz D, Turkmenoglu TT, Kabukcuoglu F (2009) The expression of p63 and p53 in keratoacanthoma and intraepidermal and invasive neoplasms of the skin. Pathol Res Pract 205: 589–594. 19577853 10.1016/j.prp.2009.01.010

[18] Mitschele T, Diesel B, Friedrich M, Meineke V, Maas RM, et al (2004) Analysis of the vitamin D system in basal cell carcinomas (BCCs). Lab Invest 84: 693–702. 15077124 10.1038/labinvest.3700096

[19] Reichrath J, Rafi L, Rech M, Mitschele T, Meineke V, et al (2004) Analysis of the vitamin D system in cutaneous squamous cell carcinomas. J Cutan Pathol 31: 224–231. 14984574 10.1111/j.0303-6987.2003.00183.x

[20] Langberg M, Rotem C, Fenig E, Koren R, Ravid A (2009) Vitamin D protects keratinocytes from deleterious effects of ionizing radiation. Br J Dermatol 160: 151–161. 18717671 10.1111/j.1365-2133.2008.08797.x

[21] Pan L, Matloob AF, Du J, Pan H, Dong Z, et al (2010) Vitamin D stimulates apoptosis in gastric cancer cells in synergy with trichostatin A/sodium butyrate-induced and 5-aza-2'-deoxycytidine-induced PTEN upregulation. Febs J 277: 989–999. 20089040 10.1111/j.1742-4658.2009.07542.x

[22] Benavides F, Oberyszyn TM, VanBuskirk AM, Reeve VE, Kusewitt DF (2009) The hairless mouse in skin research. J Dermatol Sci 53: 10–18. 18938063 10.1016/j.jdermsci.2008.08.012 PMC2646590

[23] Arbour NC, Prahl JM, DeLuca HF (1993) Stabilization of the vitamin D receptor in rat osteosarcoma cells through the action of 1,25-dihydroxyvitamin D3. Mol Endocrinol 7: 1307–1312. 8264662 10.1210/mend.7.10.8264662

[24] Backman SA, Ghazarian D, So K, Sanchez O, Wagner KU, et al (2004) Early onset of neoplasia in the prostate and skin of mice with tissue-specific deletion of Pten. Proc Natl Acad Sci U S A 101: 1725–1730. 14747659 10.1073/pnas.0308217100 PMC341836

[25] Suzuki A, Itami S, Ohishi M, Hamada K, Inoue T, et al (2003) Keratinocyte-specific Pten deficiency results in epidermal hyperplasia, accelerated hair follicle morphogenesis and tumor formation. Cancer Res 63: 674–681. 12566313

[26] Fukushima H, Koga F, Kawakami S, Fujii Y, Yoshida S, et al (2009) Loss of DeltaNp63alpha promotes invasion of urothelial carcinomas via N-cadherin/Src homology and collagen/extracellular signal-regulated kinase pathway. Cancer Res 69: 9263–9270. 19934319 10.1158/0008-5472.CAN-09-1188

[27] Urist MJ, Di Como CJ, Lu ML, Charytonowicz E, Verbel D, et al (2002) Loss of p63 expression is associated with tumor progression in bladder cancer. Am J Pathol 161: 1199–1206. 12368193 10.1016/S0002-9440(10)64396-9 PMC1867279

[28] Ming M, Han W, Maddox J, Soltani K, Shea CR, et al (2010) UVB-induced ERK/AKT-dependent PTEN suppression promotes survival of epidermal keratinocytes. Oncogene 29: 492–502. 19881543 10.1038/onc.2009.357 PMC2813408

[29] Chung M, Lee J, Terasawa T, Lau J, Trikalinos TA (2011) Vitamin D with or without calcium supplementation for prevention of cancer and fractures: an updated meta-analysis for the U.S. Preventive Services Task Force. Ann Intern Med 155: 827–838. 22184690 10.7326/0003-4819-155-12-201112200-00005

[30] Eide MJ, Johnson DA, Jacobsen GR, Krajenta RJ, Rao DS, et al (2011) Vitamin D and nonmelanoma skin cancer in a health maintenance organization cohort. Arch Dermatol 147: 1379–1384. 21844426 10.1001/archdermatol.2011.231

[31] Tang JY, Parimi N, Wu A, Boscardin WJ, Shikany JM, et al (2010) Inverse association between serum 25(OH) vitamin D levels and non-melanoma skin cancer in elderly men. Cancer Causes Control 21: 387–391. 19921445 10.1007/s10552-009-9470-4 PMC2835729

[32] van Dam RM, Huang Z, Giovannucci E, Rimm EB, Hunter DJ, et al (2000) Diet and basal cell carcinoma of the skin in a prospective cohort of men. Am J Clin Nutr 71: 135–141. 10617958 10.1093/ajcn/71.1.135

[33] Liefer KM, Koster MI, Wang XJ, Yang A, McKeon F, et al (2000) Down-regulation of p63 is required for epidermal UV-B-induced apoptosis. Cancer Res 60: 4016–4020. 10945600

[34] Ogawa E, Okuyama R, Egawa T, Nagoshi H, Obinata M, et al (2008) p63/p51-induced onset of keratinocyte differentiation via the c-Jun N-terminal kinase pathway is counteracted by keratinocyte growth factor. J Biol Chem 283: 34241–34249. 18849344 10.1074/jbc.M804101200 PMC2662236

[35] Westfall MD, Joyner AS, Barbieri CE, Livingstone M, Pietenpol JA (2005) Ultraviolet radiation induces phosphorylation and ubiquitin-mediated degradation of DeltaNp63alpha. Cell Cycle 4: 710–716. 15846104 10.4161/cc.4.5.1685

[36] Rebel HG, Bodmann CA, van de Glind GC, de Gruijl FR (2012) UV-induced ablation of the epidermal basal layer including p53-mutant clones resets UV carcinogenesis showing squamous cell carcinomas to originate from interfollicular epidermis. Carcinogenesis 33: 714–720. 22227037 10.1093/carcin/bgs004

[37] Zineb R, Zhor B, Odile W, Marthe RR (1998) Distinct, tissue-specific regulation of vitamin D receptor in the intestine, kidney, and skin by dietary calcium and vitamin D. Endocrinology 139: 1844–1852. 9528970 10.1210/endo.139.4.5903

[38] Fortmann SP, Burda BU, Senger CA, Lin JS, Whitlock EP (2013) Vitamin and mineral supplements in the primary prevention of cardiovascular disease and cancer: An updated systematic evidence review for the U.S. Preventive Services Task Force. Ann Intern Med 159: 824–834. 24217421 10.7326/0003-4819-159-12-201312170-00729

[39] Kraemer A, Chen IP, Henning S, Faust A, Volkmer B, et al (2013) UVA and UVB irradiation differentially regulate microRNA expression in human primary keratinocytes. PLoS One 8: e83392. 24391759 10.1371/journal.pone.0083392 PMC3877020

[40] Syed DN, Afaq F, Mukhtar H (2012) Differential activation of signaling pathways by UVA and UVB radiation in normal human epidermal keratinocytes. Photochem Photobiol 88: 1184–1190. 22335604 10.1111/j.1751-1097.2012.01115.x PMC3445260

[41] Thomas-Ahner JM, Wulff BC, Tober KL, Kusewitt DF, Riggenbach JA, et al (2007) Gender differences in UVB-induced skin carcinogenesis, inflammation, and DNA damage. Cancer Res 67: 3468–3474. 17389759 10.1158/0008-5472.CAN-06-3798

[42] Burns EM, Tober KL, Riggenbach JA, Schick JS, Lamping KN, et al (2013) Preventative topical diclofenac treatment differentially decreases tumor burden in male and female Skh-1 mice in a model of UVB-induced cutaneous squamous cell carcinoma. Carcinogenesis 34: 370–377. 23125227 10.1093/carcin/bgs349 PMC3564442

[43] Krishnan AV, Swami S, Feldman D (2013) Equivalent anticancer activities of dietary vitamin D and calcitriol in an animal model of breast cancer: importance of mammary CYP27B1 for treatment and prevention. J Steroid Biochem Mol Biol 136: 289–295. 22939886 10.1016/j.jsbmb.2012.08.005 PMC3554854

[44] Morris RJ, Tryson KA, Wu KQ (2000) Evidence that the epidermal targets of carcinogen action are found in the interfollicular epidermis of infundibulum as well as in the hair follicles. Cancer Res 60: 226–229. 10667563

[45] Faurschou A, Haedersdal M, Poulsen T, Wulf HC (2007) Squamous cell carcinoma induced by ultraviolet radiation originates from cells of the hair follicle in mice. Exp Dermatol 16: 485–489. 17518988 10.1111/j.1600-0625.2007.00551.x

[46] Lutzow-Holm C, De Angelis P, Grosvik H, Clausen OP (1993) 1,25-Dihydroxyvitamin D3 and the vitamin D analogue KH1060 induce hyperproliferation in normal mouse epidermis. A BrdUrd/DNA flow cytometric study. Exp Dermatol 2: 113–120. 8162327 10.1111/j.1600-0625.1993.tb00018.x

[47] Fleet JC, Gliniak C, Zhang Z, Xue Y, Smith KB, et al (2008) Serum metabolite profiles and target tissue gene expression define the effect of cholecalciferol intake on calcium metabolism in rats and mice. J Nutr 138: 1114–1120. 18492843 10.1093/jn/138.6.1114 PMC2542586

[48] Burns EM, Tober KL, Riggenbach JA, Kusewitt DF, Young GS, et al (2013) Extended UVB Exposures Alter Tumorigenesis and Treatment Efficacy in a Murine Model of Cutaneous Squamous Cell Carcinoma. J Skin Cancer 2013: 246848. 24286011 10.1155/2013/246848 PMC3826430

